# The revelation of genomic breed composition using target capture sequencing: a case of *Taxodium*

**DOI:** 10.48130/forres-0024-0031

**Published:** 2024-10-08

**Authors:** Zhitong Han, Yangkang Chen, Xiaogang Dai, Chaoguang Yu, Jiajin Cheng, Jialiang Li, Kangshan Mao

**Affiliations:** 1 Key Laboratory of Bio-Resource and Eco-Environment of Ministry of Education, College of Life Sciences, State Key Laboratory of Hydraulics and Mountain River Engineering, Sichuan University, Chengdu 610065, Sichuan, PR China; 2 State Key Laboratory of Tree Genetics and Breeding, Co-Innovation Center for the Sustainable Forestry in Southern China, College of Forestry, Nanjing Forestry University, Nanjing 210037, PR China; 3 Jiangsu Key Laboratory for the Research and Utilization of Plant Resources, Institute of Botany, Jiangsu Province and Chinese Academy of Sciences (Nanjing Botanical Garden Mem. Sun Yat-Sen), Nanjing 210014, PR China

**Keywords:** *Taxodium*, Hybridization, Target sequence capture, SNPs

## Abstract

*Taxodium* plants have good flood tolerance and thus were introduced into China from North America in the early 1900s. The subsequent decades of cross-breeding experiments within *Taxodium* have produced many new hybrid cultivars in China while also creating confusion in the genetic background of *Taxodium* plants. In the present study, target capture sequencing-derived SNP markers were used to reveal the genomic composition of different species and cultivars of *Taxodium*. The results unraveled the phylogenetic relationship within *Taxodium* and suggested the possibility of recent interspecific natural hybridization events. The introduced (Chinese) population is genetically similar to the native (North American) population, thus providing genetic evidence for historical introductions of *Taxodium*. Hybrid categories of different cultivars of *Taxodium* hybrid 'Zhongshanshan' were further identified, and their differences in parentage were revealed. Collectively, the findings provide evidence for understanding the genetics and hybridization of *Taxodium* and shed light on the future breeding and cultivation of cultivars with great ecological and economic potential.

## Introduction

Native North American genus *Taxodium*, comprising three currently known species, *T. ascendens* Brongn., *T. distichum* (L.) Rich., and *T. mucronatum* Ten., was introduced into China in the early 20^th^ century for use in forestry^[1−5]^. Since the 1960s, Chinese researchers have been crossbreeding within *Taxodium* and have produced a succession of interspecific hybrid varieties named by *Taxodium* hybrid 'Zhongshanshan' (ZSS)^[6]^. The inherent tolerance of *Taxodium* to environmental stresses including flooding and salt^[7−9]^, and decades of selective breeding have combined to shape the ecological adaptability of ZSS, which has strong resistance to a wide range of environmental stresses including wind, perennial flood, salinization, and alkalinization^[10,11]^. ZSS has therefore been widely cultivated in wetland, riverbanks, and the coastal floodplains of eastern China for flood control and landscaping^[10,12]^.

In recent years, due to the great ecological and economic potential of *Taxodium*, an increased interest in its genetic background and gene functions has emerged. A few studies have used bioinformatics and molecular biology approaches to identify and characterize specific genes inherited by *Taxodium* that play important roles in the tolerance of diverse environmental stresses, and to reveal the expression and regulation of these genes in response to environmental changes^[13,14]^. In contrast, the genetic background and interspecific phylogenetic relationships within *Taxodium* have been rarely studied, leaving a controversial definition of species and varieties within the genus. Despite Tsumura et al.^[15]^ used cleaved amplified polymorphic sequences (CAPS) markers to study phylogenetic relationships among North American taxa, introduced hybrid *Taxodium* species/variants in China remain overshadowed by their more than 100-year history of introduction and morphological similarity. Previous studies on *Taxodium* cultivated in China have sought to ascertain the genetic background within the genus adopted the random amplified polymorphic DNA (RAPD), sequence-related amplified polymorphism (SRAP) and comparative chloroplast genomics^[1,16,17]^. Zheng et al.^[6]^ applied electrochemical fingerprints to identify *Taxodium* taxa and derived hybrid progenies. Among these studies, cultivars of ZSS (including 'ZSS301', 'ZSS302', 'ZSS401', and 'ZSS405') have been extensively researched. The common parentage of these cultivars was from the colony of Chinese introduced *T. mucronatum* (ITM), which is considered to be the progeny of cuttings from North American *T. mucronatum* and has a long history of cultivation in China, but has scarcely been studied. In addition, the genetic relationship between the Chinese and North American *Taxodium* plants need to be further clarified. Unlike North American populations where the Chinese populations originate, the latter may have lower genetic diversity due to founder effects and inbreeding, which in turn, obstructs its expansion and forestry applications.

Nevertheless, in-depth studies applying efficient sequencing techniques and genome-wide markers are rare in *Taxodium* spp., limiting the understanding of their taxonomy and genomic breed compositions. In the present study, the aim is to provide new insights into the following questions: (1) What is the genetic component of ZSS? (2) What are the phylogenetic relationships within the genus *Taxodium*? (3) How is the ecological suitability of *Taxodium* in China? The target capture sequencing method was applied to sequence the genome-wide exomes which are subsequently called nuclear and chloroplast SNPs, separately. The SNPs were used to develop multiple population genetic and evolutionary analyses, which it is believed will cast light on the genomic breed composition and kinship of *Taxodium*. Species Distribution Modeling (SDM) results were integrated to facilitate the understanding of the ecological suitability of the genus, which may provide a reference for future introduction and cultivation.

## Materials and methods

### Plant materials

The leaves of 31 individuals were sampled and numbered into seven groups, including ITM (*T. mucronatum* introduced in China), Mxg (*T. mucronatum* native to Mexico), ZSS (*T.* hybrid 'Zhongshanshan'), Lys (*T. distichum*), Cs (*T. ascendens*), Ss (*Glyptostrobus pensilis*) and Ls (*Cryptomeria fortunei*) (Supplemental Table S1). Most of these samples were collected in 2019 in the middle and lower Yangtze River Plain (China), the others (all belonging to Mxg) were provided by the Royal Botanic Garden, Edinburgh, UK. The Mxg group was collected to compare the genetic composition with the ITM group, thereby verifying their relationship. Four individuals were included in the ZSS group, each of a different cultivar ('Zhongshanshan111', 'Zhongshanshan302', 'Zhongshanshan401', 'Zhongshanshan405'), were used to study the genetic background of the ZSS group and to compare the hybrid composition of the different cultivars. These cultivars of ZSS are morphologically similar but vary in genetic backgrounds, and they are reported as F1 generations that are crossed between different species^[6]^. The Ls group was collected because *C. fortunei* may have been involved in a controversial intergeneric hybrid, thus becoming a potential donor of genetic material to the ZSS^[3,18]^. The Ss group was collected as an outgroup in the phylogenetic analysis. All samples were kept at −80°C until DNA extraction.

### Data collection, reads mapping, and SNP calling

The targeted region was captured using the NimbleGen SeqCap EZ probes which was designed by Li et al.^[19]^, and sequenced following the standard Illumina library construction protocol (Illumina, San Diego, California, USA). The data volume of each sample was 1,000 to 6,000 M according to the species.

The quality of Illumina raw reads was controlled *via* Trimmomatic version 0.36^[20]^. Then BWA version 0.7.17^[21]^ with default parameters was used to align the filtered reads to the transcriptome and the chloroplast genome of *T. mucronatum*, obtained from 1,000 plants project^[22]^ and NCBI separately. SAMtools version 1.9^[23]^ was used to convert the file format. Duplicates produced by PCR were marked by Picard version 2.20.3^[24]^. The variants were called using HaplotypeCaller implemented in GATK version 4.1.2^[25]^ for each sample. After combining the GVCF files, genotypes and SNPs were called using GATK-GenotypeGVCFs and GATK-SelectVariants. Finally, the official guide of GATK was used, SNPs were filtered by GATK-VariantFiltration, with parameters that exclude SNPs 'QD < 2.0; QUAL < 30.0; SOR > 3.0; FS > 60.0; MQ < 40.0; MQRankSum < −12.5; ReadPosRankSum < −8.0'. VCFtools version1.9^[26]^ was used to further filter the remaining SNPs by Minor Allele Frequency (MAF) and missing data, the parameters were set as '--max-alleles 2 --min-alleles 2 --max-missing 0.8 --maf 0.05 --minDP 3 --maxDP 1000'.

### Population genetic analysis

Nuclear and chloroplast pairwise distances between ITM and other taxa were computed under both the Tajima-Nei model and the Maximum Composite Likelihood method implemented in MEGA version 10.1.5^[27]^, the values of which are shown as the average standard of each taxon (Supplemental Fig. S1). Then a model-based evolutionary clustering analysis was conducted *via* ADMIXTURE version 1.3.0^[28]^ to analyze population genetic structure using nuclear SNPs. In the Principal Component Analysis (PCA), PLINK version 1.9^[29]^ and VCFtools version1.9^[26]^ were used to produce PCA files using the nuclear data, and SMARTPCA implemented in EIGENSTART version 6.1.3^[30]^ to conduct the analysis.

### Phylogenetic and neighbour-net inference

RAxML version 8.0.0^[31]^ was used to build maximum likelihood (ML) phylogenetic trees with the substitution model GAMMA for both the nuclear SNPs and the chloroplast SNPs. The clades' relative robustness was estimated by performing 1,000 bootstrap replicates based on which a 95% confidence network was constructed. Based on the nuclear ML phylogeny, the divergence times of *Taxodium* and related genera were further estimated using the Bayesian sequential-subtree dating approach^[32]^, which was implemented in PAML version 4.10.7^[33]^. The divergence times estimates was incorporated with three calibration points^[34]^, each for a node between genus (Supplemental Table S2). To compare the result from RAxML, NINJA^[35]^ was also used to build a neighbour-joining (NJ) tree with chloroplast SNPs. To investigate the hybridization events in the cultivation history of ITM, the Neighbor network method implemented in SplitsTree version 4.15.1^[36]^ was applied to reconstruct reticulate networks with nuclear SNPs.

### Genetic inference with selected SNP panels

To further discover diagnostic SNPs, the population genetics differentiation (*F*_ST_) between *T. distichum* and *T. ascendens* was calculated for each SNP by VCFtools version 1.9^[26]^. The hybrid proportion was quantified by Detection of Recent Hybridization (DRH) analysis^[37]^. This analysis detects hybrids by genotyping individuals at multiple loci and calculating two metrics: allelic dosage (fraction of alleles from one parental source) and observed heterozygosity (*Ho*). It plots these values and uses confidence regions to classify individuals into genealogical groups including F1 hybrids, backcrosses, or parentals based on expected patterns under Mendelian inheritance. Significance is inferred when confidence regions don't overlap, indicating distinct hybrid classes from parental populations. Following the instruction from Vonholdt et al.^[37]^, a 24-SNP panel and a 100-SNP panel were subset for the DRH analysis. Both panels were determined by *Fst* (SNPs with the highest *Fst* values were kept), which adequately represents the genetic divergence among populations. R version 4.0.5^[38]^ was then used to calculate the average number of non-reference (non-*T. distichum*) alleles of each locus and the fraction of each individual's heterozygous loci, and then present them orthogonally. In this way, the parents in the hybrid event should be around the base angles, one on each side, and the hybrid F1 should be around the vertex angle. To cross-validate the hybrid stages of the four ZSS samples, NewHybrids Version 2.0^[39]^ was used to compute the posterior distribution that each falls into different categories using the 100-SNP panel. This test was conducted on all samples, as well as separate ZSS and its parents. The output was then plotted using the package hybriddetective Version 0.1.0.9000^[40]^, which was implemented in R.

### Species distribution modeling

Spatial distribution data for the three *Taxodium* species, limited to their native habitats in North America, were downloaded from the Global Biodiversity Information Facility (GBIF)^[41]^. The distribution points were then sparsified to prevent overfitting of the model. The Nearest Neighbor Distance (NND) between retained distribution points were set to be > 5 km, and the thinning process was performed for 100 iterations using the package spThin^[42]^ implemented in R. The data was finally filtered to retain 367 distribution points, including 47 for *T. ascendens*, 284 for *T. distichum,* and 36 for *T. mucronatum*. Climate data was downloaded from WorldClim 1.4^[43]^, including periods of current (1960−1990), future (2050 and 2070 under RCP2.6 scenario) and Last Glacial Maximum, with a resolution of 2.5' (5 km × 5 km). Distribution modeling was then conducted in Maxent version 3.4.1^[44]^ following the methods section of Qin et al.^[45]^. All 19 climate variables were first put into the model for data wrangling, the Jacknife method was used to calculate the contribution of each variable, and variables removed with r ≥ 0.7 Pearson correlation coefficient and a low contribution. (See Supplemental Fig. S2 and Supplemental Table S3 for the performance and contribution of each ecological factor). The distribution points of *Taxodium* were subset into test (25%) and training (75%) sets, which were imported into the Maxent for 500 'Subsample' iterations along with the filtered climate variables. The rule for thresholding was selected as 'Maximum training sensitivity plus specificity', and other parameters were set to default values. After evaluating the performance of the model using the area under the receiver operating characteristic curve (AUC), Maximum training sensitivity plus specificity (MTSS) was adopted, Cloglog threshold, implemented in Maxent, to reclassify the habitat suitability: unsuitable habitat (< 1*MTSS); barely suitable habitat (1*MTSS–2*MTSS); suitable habitat (2*MTSS–3*MTSS); highly suitable habitat (3*MTSS <).

## Results

### SNPs calling

After reads mapping, variants calling, and filtration for all samples from seven populations, 2,752,534 nuclear SNPs and 6,901 chloroplast SNPs were revealed. For hybrid stage delimitation and genetic components quantification, the SNPs were called again for all taxon except for *G. pensilis*, *M. glyptostroboides*, and *C. fortunei* and obtain 28,142 SNPs. The scaled overall heterozygosity for each *Taxodium* population was calculated based on the shared SNP markers (see Supplemental Fig. S3). In detection of recent hybridization (DRH) analysis and hybrid stage analysis, 24 and 100 SNP panels with the highest contribution on *F_st_* statistic among populations were further filtered out.

### Genetic distances and population genetic structure

**Figure 1:**
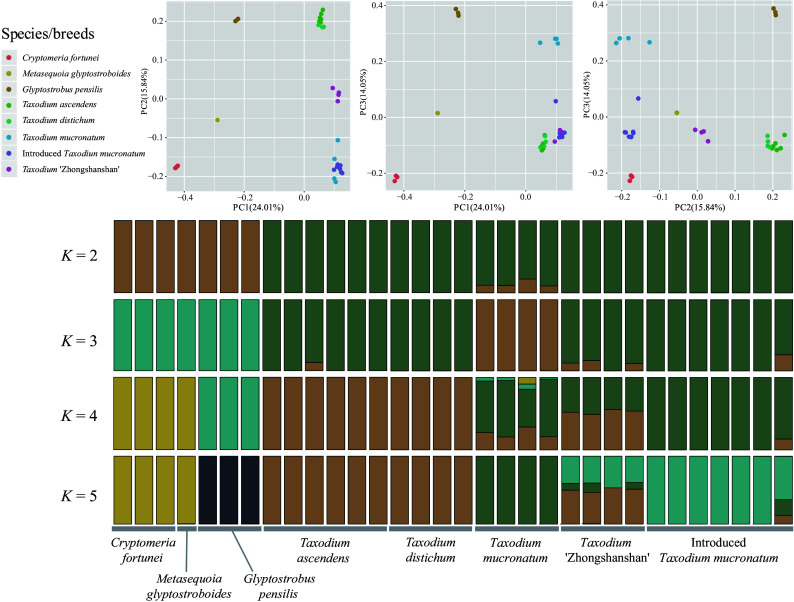
Population structure analysis with PCA and ADMIXTURE. Principal component analysis (PCA) of the seven taxa involved for 2,752,534 nuclear SNPs. The first and the second eigenvectors separated *G. pensilis*, *M. glyptostroboides*, and *C. fortunei* from the *Taxodium* (including ITM; *p* = 0.0252, 0.0113 and 0.0004 separately, Tracy-Widom test). The third eigenvector segregated each species/breed within *Taxodium* (*p* = 3.47026e-08). Genetic clustering of species and cultivars inferred by ADMIXTURE. Simulations were set at 1,000 bootstraps. Each individual is represented by a thin vertical bar, which is partitioned into K-colored segments and represents the individual affiliation to each cluster (*K* is set from 2 to 10). Delta *K* = 2 and *K* = 4 are the two peak values according to cross-validation analysis.

According to the Admixture and cross-validation analysis result, *K* = 2 is the best supported model, and *K* = 4 is the second. When *K* = 2, all *Taxodium* samples (including cultivars) were clearly distinguished from the outgroups (*Cryptomeria-Glyptostrobus* cluster) (Fig. 1). When *K* = 4, new clusters are subdivided within the *Taxodium*, showing ITM and *T. mucronatum* as an integrated cluster and that ZSS shares both SNPs from the *Taxodium distichum* cluster and ITM cluster. The result indicates that ITM has a similar genetic composition to the natural population of *T. mucronatum* in Oaxaca, Mexico (Mxg50J). Samples of ZSS showed distinct hybrid features under *K* = 4 and *K* = 5 models, that each of the four cultivars can be half-and-half affiliated to *T. ascendens*-*T. distichum* cluster and ITM-*T. mucronatum* cluster. Evidence was also found that partial samples of *T. mucronatum* may have experienced hybrid or genetic introgression events that present complicated genetic composition when K = 4 and 5. Individuals of *T. ascendens* and *T. distichum* have remained in the same cluster under different K values, showing high genetic similarity.

In the principal component analysis (PCA), the first principal component (PC1), which explained 24.01% of all genetic variance, separated *Cryptomeria*, *Glyptostrobus,* and *Taxodium* into three clusters. PC2, which explained 15.84% genetic variance, further separated *Taxodium* into three clusters: the *T. ascendens*-*T. distichum* cluster, the ZSS cluster, and the ITM-*T. mucronatum* cluster, with the ZSS cluster occupying an intermediate space between the other two. PC3 primarily subdivides the populations of *T. mucronatum* (Fig. 1). PCA shows a close relationship between ITM and the native *T. mucronatum*. Meanwhile, ZSS represented a mixture of genetic components between the *T. ascendens*-*T. distichum* and *T. mucronatum* cluster, indicating former hybrid events. Consistent with the results of the genetic structure analysis above, *T. ascendens* and *T. distichum* remained highly coherent in PC space, forming a stable cluster.

### Phylogenetic analysis

**Figure 2:**
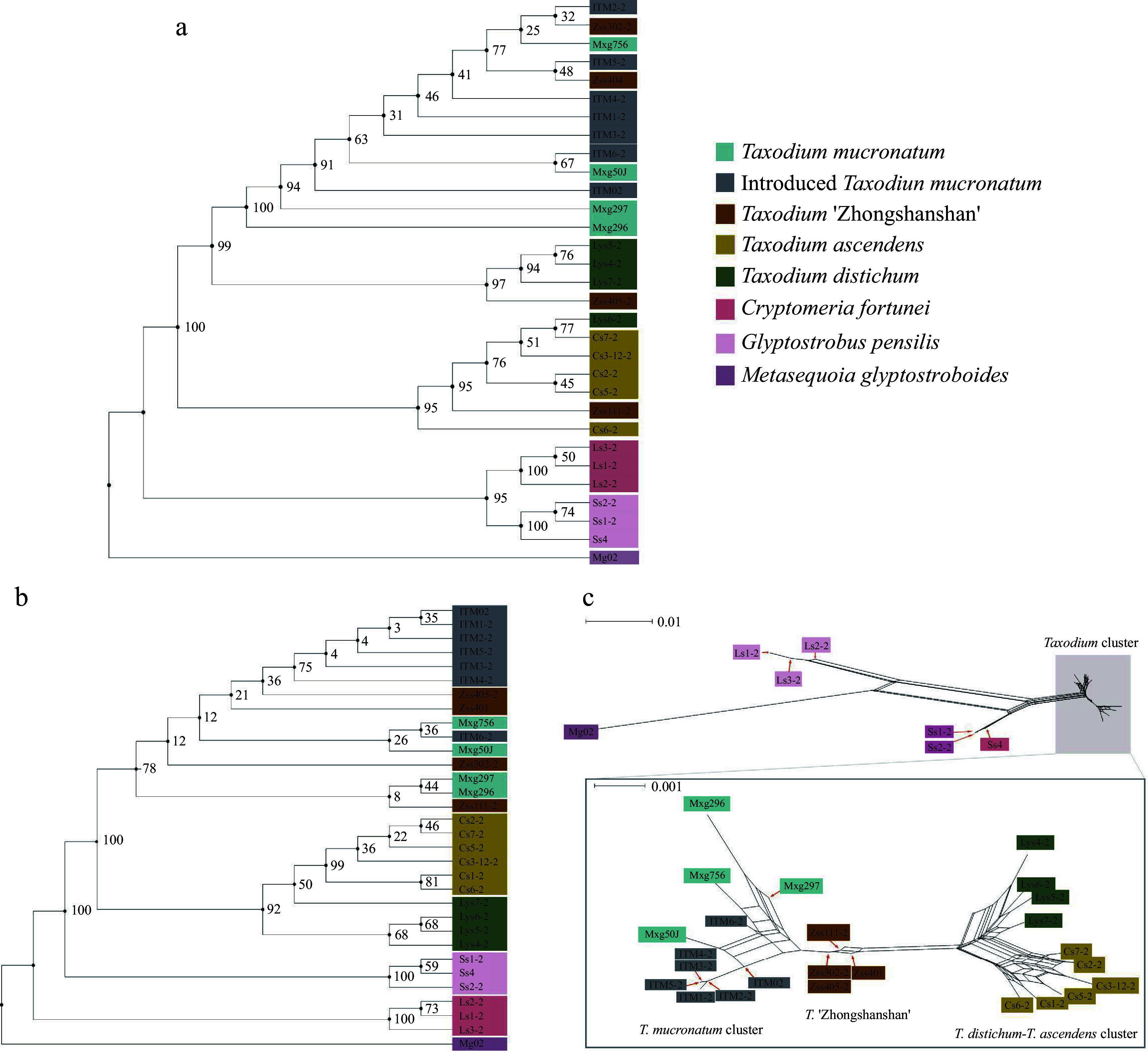
Phylogenetic and neighbour-net analyses. Colour represents the population. Branch labels are bootstrap support values from 1,000 replicates. (a) ML tree based on 6,901 chloroplast SNPs. (b) Maximum Likelihood tree based on 2,752,534 nuclear SNPs. (c) Results of neighbour-net analysis based on 2,752,534 nuclear SNPs, with a zoomed-in view of the *Taxodium* cluster in the lower half. All species and cultivars are highlighted in different colours, and the length of the lines indicates the distance among clusters/individuals.

The phylogenetic trees generated from both maximum likelihood (ML) and neighbour-joining (NJ) methods showed similar clustering information for chloroplast SNPs (Fig. 2a). Both trees split all *Taxodium* samples into three distinct clusters: ITM-*T. mucronatum*, *T. distichum*, and *T. ascendens* cluster, using *G. pensilis* and *C. fortunei* as outgroups. Since the chloroplast genomes are believed to be paternally inherited in Cupressaceae *sensu lato*^[46]^, the chloroplast phylogenetic tree indicates each individual's direct paternal parent. The ITM and native *T. mucronatum* clustered together, which confirmed the conjecture that most ITM individuals are derived from one of the earliest introduced *T. mucronatum* individuals (ITM02), introduced to mainland China in 1925. Zss302-2 (*T*. '*Zhongshanshan 302*') and Zss401 (*T*. '*Zhongshanshan 401*') are located inside ITM-*T. mucronatum* cluster, suggesting that the paternal species of them are identified as *T. mucronatum*. Similarly, since Zss405-2 (*T*. '*Zhongshanshan 405'*) and Zss111-2 (*T*. '*Zhongshanshan 111*') clustered with *T. distichum* and *T. ascendens* separately, their paternal parents were also indicated.

To further reveal the phylogenetic relationships within *Taxodium*, a ML tree for 2,752,534 nuclear SNPs was constructed (Fig. 2b). ITM and ZSS admixed with *T. mucronatum*, suggesting the indivisible kinship of these three species and cultivars. The result indicated that ZSS has a closer affinity to *T. mucronatum* than the other two parents, *T. distichum* and *T. ascendens*. In phylogenetic analysis, *T. distichum* and *T. ascendens* together form a monophyletic clade, rather than being sister branches to each other. The support values within this monophyletic clade are also low, with half of them showing support values ≤ 50. The neighbour-net analysis further revealed the relationship among all clusters (Fig. 2c). The plot intimated the same kinship network as the previous analyses did, with a more distinct view of the hybrid property of ZSS. Five ITM samples were derived from ITM02, confirming the parental identity of ITM02 in the initial colonization. Meanwhile, an evolutionary timescale of *Taxodium* was reconstructed and the divergence time estimation based on nuclear SNPs suggested that *Taxodium* diverged into three species in Late Paleocene to early Eocene (median ages: 62.54−52.47 Ma), with *T. mucronatum* diverged before the split of the other two species (Supplemental Fig. S4).

### Hybridization inference with representative SNPs panels

**Figure 3:**
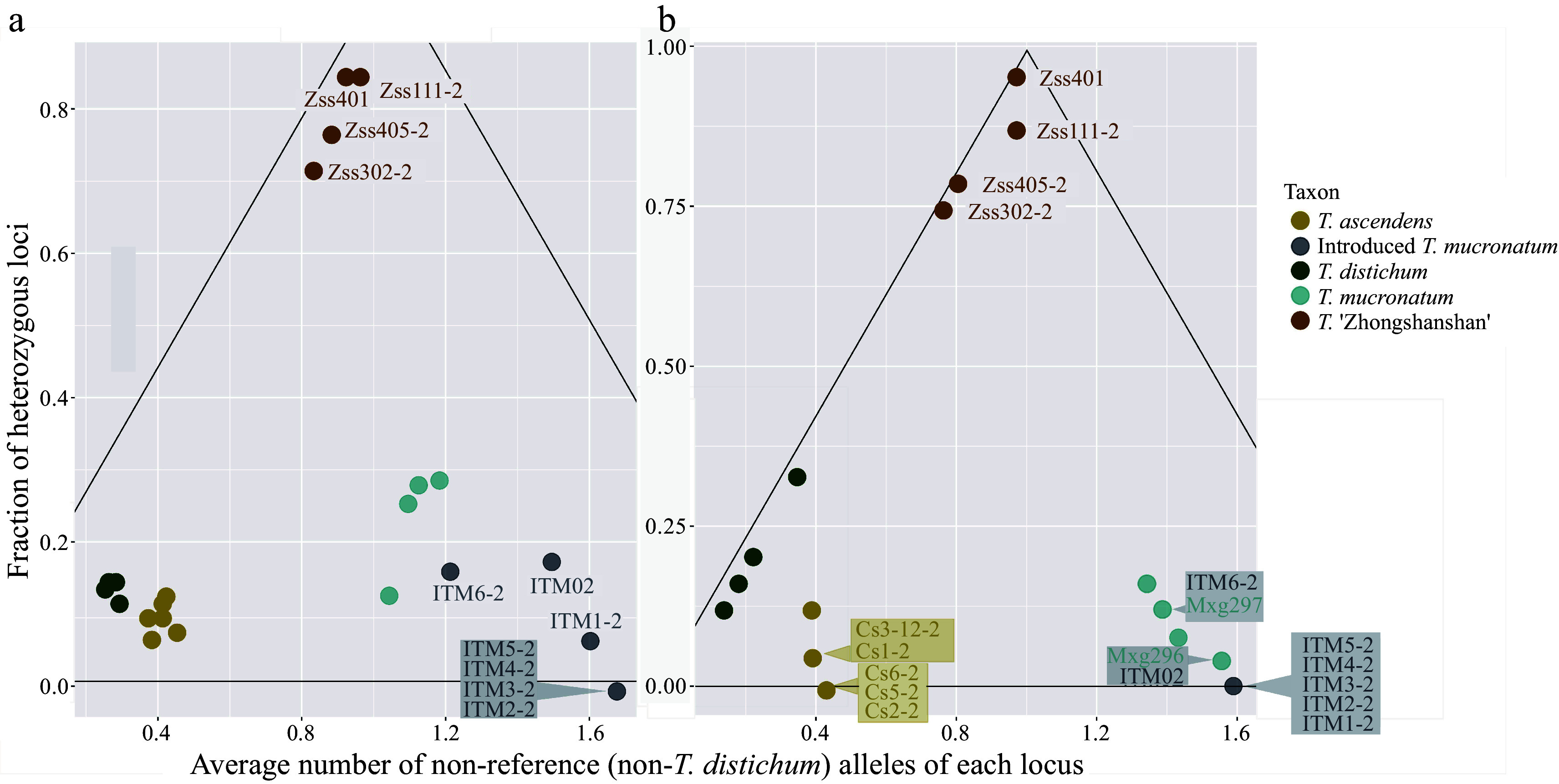
Detection of recent hybridization (DRH) analysis. Each dot represents an individual, and the colour shows the taxon. Hybrid individuals and individuals with overlapping positions are labelled. The black lines delineated the triangle formed a hybrid region, the vertex angle of which represents pure F1 generation and the base angles represent pure parents (one on each side). (a) DRH analysis based on the 100-SNP panel. (b) DRH analysis based on the 24-SNP panel.

According to the results of genetic inference, the *T. ascendens*-*T. distichum* cluster is located on one parent angle and the ITM-*T. mucronatum* cluster located on the other (Fig. 3). ZSS plants occupied the vertex angle, indicating a hybrid genetic composition of both clusters. Due to the close phylogenetic relationship of *Taxodium* species, the result may involve polymorphic markers to present both parents not purely polarized. ITM shows its attribute as part of *T. mucronatum* with even further genetic distances to the *T. ascendens*-*T. distichum* cluster than native *T. mucronatum* individuals. In both 24-SNP and 100-SNP cases, *T. mucronatum* shows a relatively large intraspecific genetic variance than *T. ascendens* and *T. distichum*.

The result of the hybrid stage inference analysis reveals the posterior probability that each sample belongs to each hybrid stage (Supplemental Table S4). Most of the individuals have a relatively high posterior probability that supports the category division. *T. ascendens* and *T. distichum* are categorized as 'parent 1'. However, this cluster cannot be subdivided based on current analysis because of the similarity of genetic composition between them. Two *T. mucronatum* individuals (Mxg50J, Mxg756) can be assigned to 'parent 2', but the other two possess a relatively large probability of being 'back cross 2' (Mxg296, Mxg297). This can be attributed to the complex genetic variation within the taxon. All four ZSS samples were assigned to 'F1, with a concrete support rate (Supplemental Fig. S5).

### Species distribution modeling inference

**Figure 4:**
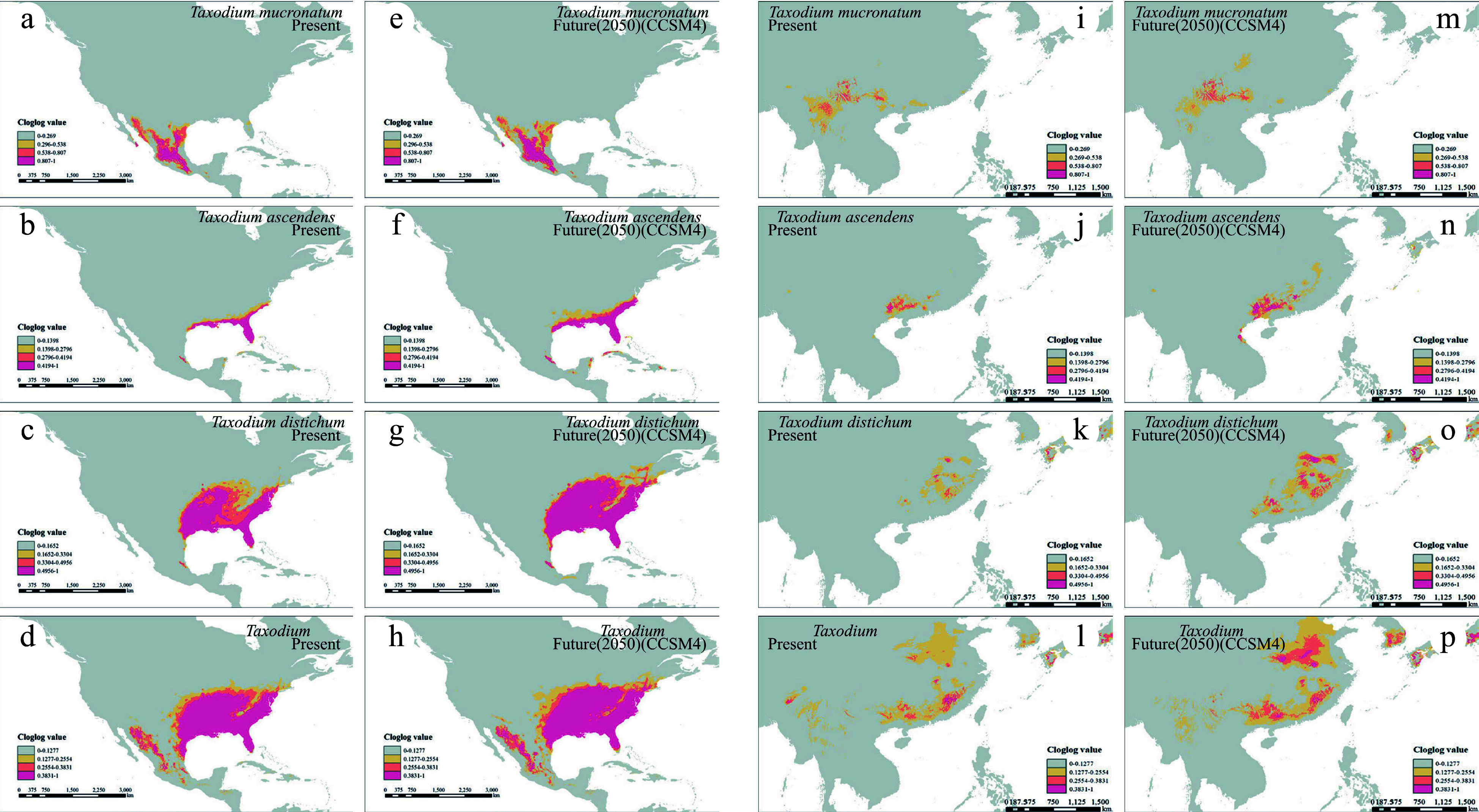
Species distribution modeling of the three *Taxodium* species in present and future 2050. (a)−(h) shows the native distribution of *Taxodium* species. (a)−(d) present the present distribution, and (e)−(h) for future 2050. Order in rows (a)−(d) represents *T. mucronatum*, *T. ascendens*, *T. distichum*, and the whole genus, respectively. The identical models' output was symmetrically projected to east coast Asia (i)−(p).

The potential distributions of the three *Taxodium* species were inferred in both North America and East Asia under present, past, and future climate scenarios (Fig. 4 & Supplemental Fig. S6). The models for all three species showed good performance in testing, with all AUC values > 0.98 (Supplemental Fig. S7). The results indicate that the suitable distribution areas (suitability score ≥ 1*MTSS) of all native North American populations expand from present to 2050 (76.8% for *T. ascendens*, 27.7% for *T. distichum* and 12.7% for *T. mucronatum*). The distribution areas in China are also simulated to have substantially increases in the cases of *T. ascendens* (106%) and *T. distichum* (109%), while the distribution of *T. mucronatum* will shrink by 10.2% in China.

## Discussion

### Genetic composition and phylogeny

The present phylogenetic analyses revealed that all samples can be divided into three clusters based on both nuclear SNPs and chloroplast SNPs: *Glyptostrobus*, *Cryptomeria*, and *Taxodium*. This result coordinates with the previous taxonomy that they represent three separate genera of Cupressaceae *sensu lato*^[46,47]^. Furthermore, the phylogenetic results of nuclear SNPs indicated a closer kinship between *T. distichum* and *T. ascendens* than *T. distichum* and *T. mucronatum*, which is consistent with previous studies^[15,48]^. Nevertheless, the chloroplast phylogeny supports a sister relationship between *T. distichum* and *T. mucronatum*, which agrees with previous studies based on the whole chloroplast genome^[1]^. Two possible explanations are presented here: First, as have been mentioned in the results, the *Taxodium* may have experienced recent natural hybrid events, which requires the collection samples to be as broad and diverse as possible in further experiments; second, the probe used in the present study are designed based on RNA sequences^[19]^, which reflect exome status. *T. distichum* and *T. ascendens* may have a close relationship in the exome region — referring to their similarity in morphological traits — but the three species have experienced incomplete lineage sorting due relatively short internode branch length between the most recent common ancestor of the three species and that of *T. distichum* and *T. ascendens*, and hence chloroplast genome, which represent a single locus on a whole, supporting a different phylogeny^[49,50]^. It is therefore believed that hybridization between *T. distichum*-*T. ascendens* cluster and *T. mucronatum*, rather than hybridization between *T. distichum* and *T. ascendens*, will be more valuable in forestry and possess higher ecological potential because they have rather far genetic distance, consistent with the current hybrid strategies of ZSS. Efforts to identify evolutionary relationships and genetic distances between these two species will require future population genetics studies that include more (for example, hundreds of) samples from different populations.

The PCA, Admixture, and phylogenetic analyses supported that ZSS appears to be a mixture of *T. mucronatum* clade and *T. distichum-T. ascendens* clade. The frequency-based analysis further confirms the conclusion. ZSS has the highest heterozygosity and locates in the middle of *T. ascendens-T. distichum* and *T. mucronatum* clades in both Bayesian hybrid inference and DRH analysis, suggesting its hybrid identity. The divergence time estimates suggest that species within *Taxodium* diverged around 52−63 million years ago, which is earlier than previous chloroplast-based estimates of divergence^[34]^. Hybridisation between species that have diverged for such a long time indicates weak reproductive isolation within *Taxodium*, which is consistent with Kou et al.^[51]^ where two genera diverged ca. 46 million years ago can still hybridize with each other. Due to the paternally inherited nature of chloroplasts in *Taxodium*^[46]^, phylogenetic results based on chloroplast (paternal) and nuclear genes can reveal the parental species of hybrid individuals of ZSS. Particularly, Zss401 with *T. mucronatum* as paternity and *T. ascendens* as maternity; Zss302-2 with *T. mucronatum* as paternity and *T. distichum* as maternity; Zss111-2 with *T. ascendens* as paternity and *T. mucronatum* as maternity; Zss405-2 with *T. distichum* as paternity and *T. mucronatum* as maternity. These results are consistent with the previous identification using electrochemical fingerprints^[6]^. For other plants with the same characteristics (i.e., paternally inherited chloroplasts), similar methods can be applied to the detection and identification of other hybrid individuals (e.g., hybrid among different species in Cupressaceae), thus facilitating breeding and forestry studies.

There is sufficient evidence to declare that ITM is not a hybrid production but a clone of *T. mucronatum*, and genetic components of most ITM samples were inherited from the ITM individual ITM02. ITM was propagated artificially from cuttings of native *T. mucronatum*^[3]^, which could leave low genetic variation and high genetic similarity within the population. The uniformity of genetic components within a population is harmful and obstructive to the expansion of the population^[52,53]^. Therefore, we suggest that native *T. mucronatum* of genetic variation from different geological locations should be introduced for the consideration of further afforestation. Future studies of the worldwide genetic variance of *T. mucronatum* and identification of the source location of ITM require a broader sample collection.

### Outlook and suggestions for future cultivation

With the assumption that hybrid cultivars can inherit the ecological traits of parental species^[54]^, hybrids with *T. distichum* as a parent should be promoted more in China in the future, as *T. distichum* has the greatest expansion of potentially suitable areas (264,200 km^2^, 109%) in *Taxodium*. However, considering the varying degrees of intra-/interspecific genetic distances and nucleoplasm conflicts in phylogenetic analysis, future hybridization experiments are still necessary.

The investigation of wild germplasms and existing cultivars in genetic and ecological distribution should be prior information for future forestry studies. We suggest that High-Throughput Sequencing should be more widely applied in forestry research. For hybrid cultivars delimitation, analysis conducted on both nuclear and chloroplast levels is necessary. Nuclear SNPs for diploid samples help to polarise the genetic components to bidirectional parental information. Because of the nature of hybridization (F1) that approximately half-to-half of the chromosomes come from each parental species, it is easy to tell whether a sample is a hybrid. On the other hand, due to its characteristics of inheritance, the chloroplast genome helps to identify paternal and maternal species of hybrids. In addition, high-throughput sequencing may also help to reveal the genetic components before assessing traits or conducting new hybridization, especially for accessions whose genomic background are not known. Meanwhile, environmental-associated SNPs and genomic selection models can serve as powerful tools to predict potential adaptions and reduce uncertainty in the experiments of hybrid, thus improving the quality of future cultivars^[55]^.

## Conclusions

By analysing the chloroplast and nuclear genes of *Taxodium*, the phylogeny of species and cultivars of the genus were constructed. Based on that, the genetic background of Chinese introduced *T. mucronatum* was further explored and the genetic components of *Taxodium* hybrid 'Zhongshanshan' identified. Given the flooding resistance of *Taxodium*, the data and results generated in this study could provide valuable resources and references for forest genetics and breeding studies of the genus. The target capture sequencing approach employed can also be applied to future forestry studies to reveal the genetic background of other woody plant species and/or hybrids.

## SUPPLEMENTARY DATA

Supplementary data to this article can be found online.

## Data Availability

The datasets generated during and/or analyzed during the current study are available from the corresponding author on reasonable request. Codes and scripts used in this study can be found under: https://github.com/chenyangkang/CNN_of_chloroplast_delimitation.
