# Supplementary material for: The revelation of genomic breed composition using target capture sequencing: a case of Taxodium
Source: For Res (Fayettev). 2024 Oct 8;4:e034. doi: 10.48130/forres-0024-0031 (PMC11524225; doi:10.48130/forres-0024-0031)
Supplement: Supplementary file 1 — Supplementary data to this article can be found online. [file forres-0024-0031-S1.zip › 10.48130_forres-0024-0031-Suppl-FigureS1.pdf]

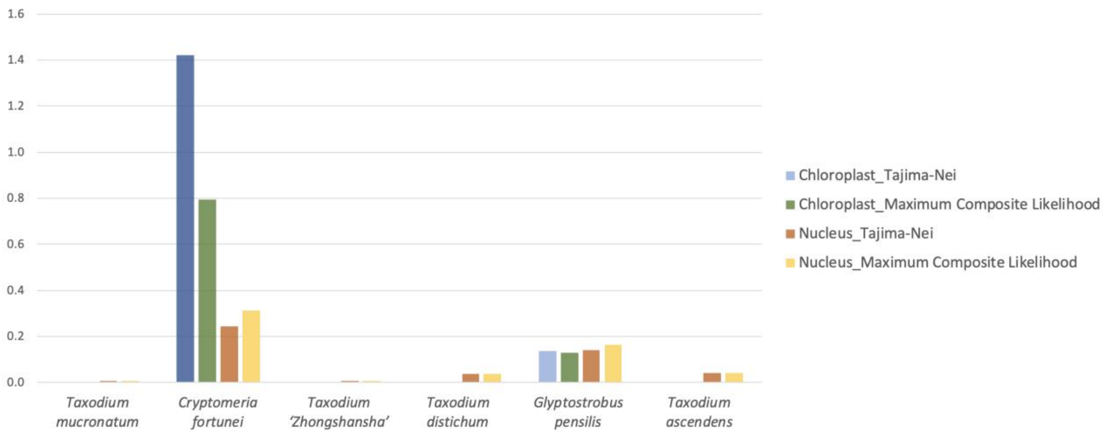

Figure S1. Pairwise distance analysis

The figure demonstrates the average genetic distance between ITM and other taxa using both Chloroplast and nuclear data under both the Tajima-Nei model and the Maximum Composite Likelihood method.
