# Supplementary material for: The revelation of genomic breed composition using target capture sequencing: a case of Taxodium
Source: For Res (Fayettev). 2024 Oct 8;4:e034. doi: 10.48130/forres-0024-0031 (PMC11524225; doi:10.48130/forres-0024-0031)
Supplement: Supplementary file 1 — Supplementary data to this article can be found online. [file forres-0024-0031-S1.zip › 10.48130_forres-0024-0031-Suppl-FigureS5.pdf]

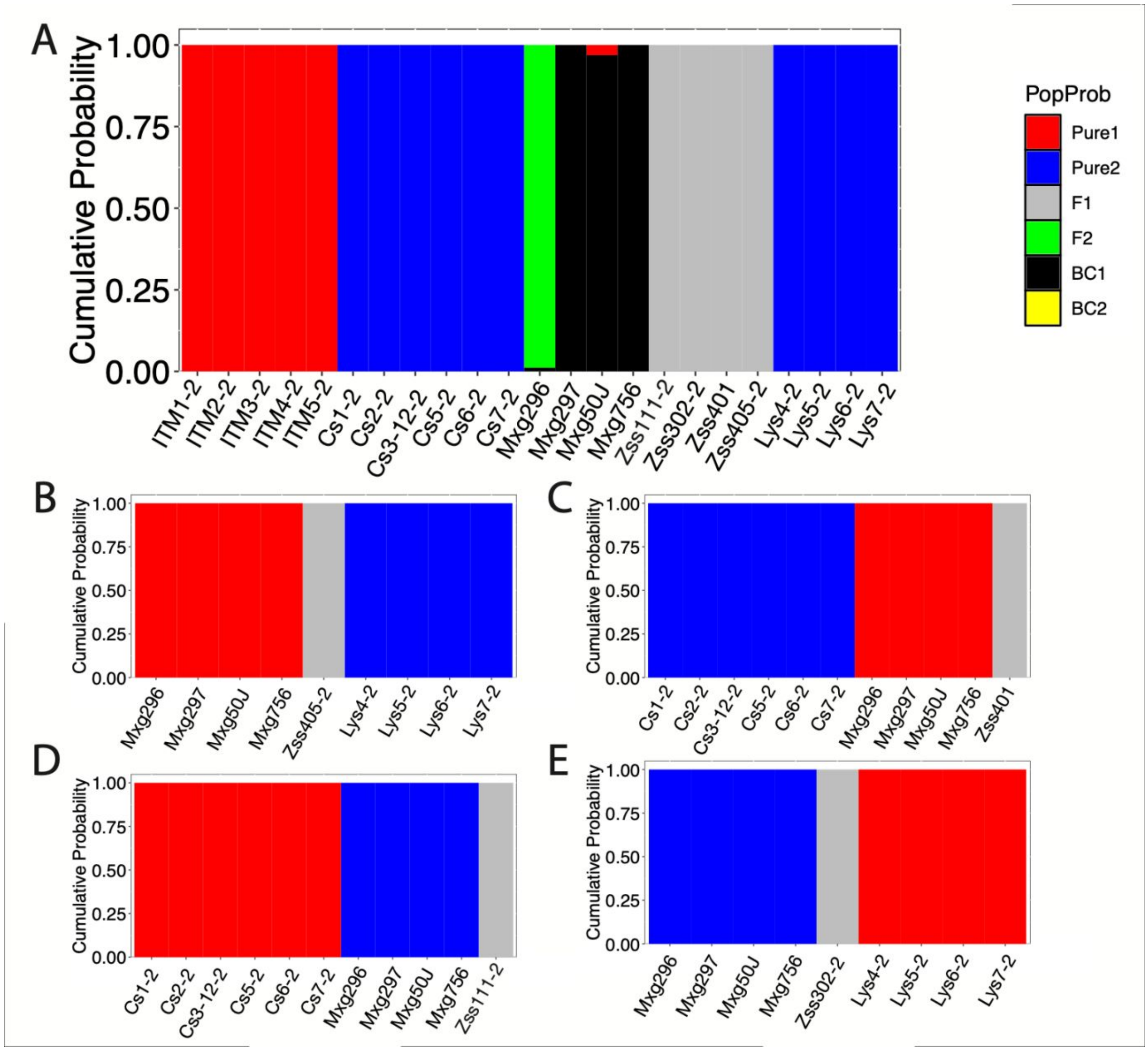

Figure S5. Hybrid stages infered by NewHybrids, ZSS samples were identified to be “F1” in all subsets. (A) All collected samples; (B) sample ‘Zss 405-2’ and its parental species; (C) sample ‘Zss 401’ and its parental species; (D) sample ‘Zss 111-2’ and its parental species; (E) sample ‘Zss 302-2’ and its parental species.
