# Supplementary material for: The revelation of genomic breed composition using target capture sequencing: a case of Taxodium
Source: For Res (Fayettev). 2024 Oct 8;4:e034. doi: 10.48130/forres-0024-0031 (PMC11524225; doi:10.48130/forres-0024-0031)
Supplement: Supplementary file 1 — Supplementary data to this article can be found online. [file forres-0024-0031-S1.zip › 10.48130_forres-0024-0031-Suppl-FigureS7.pdf]

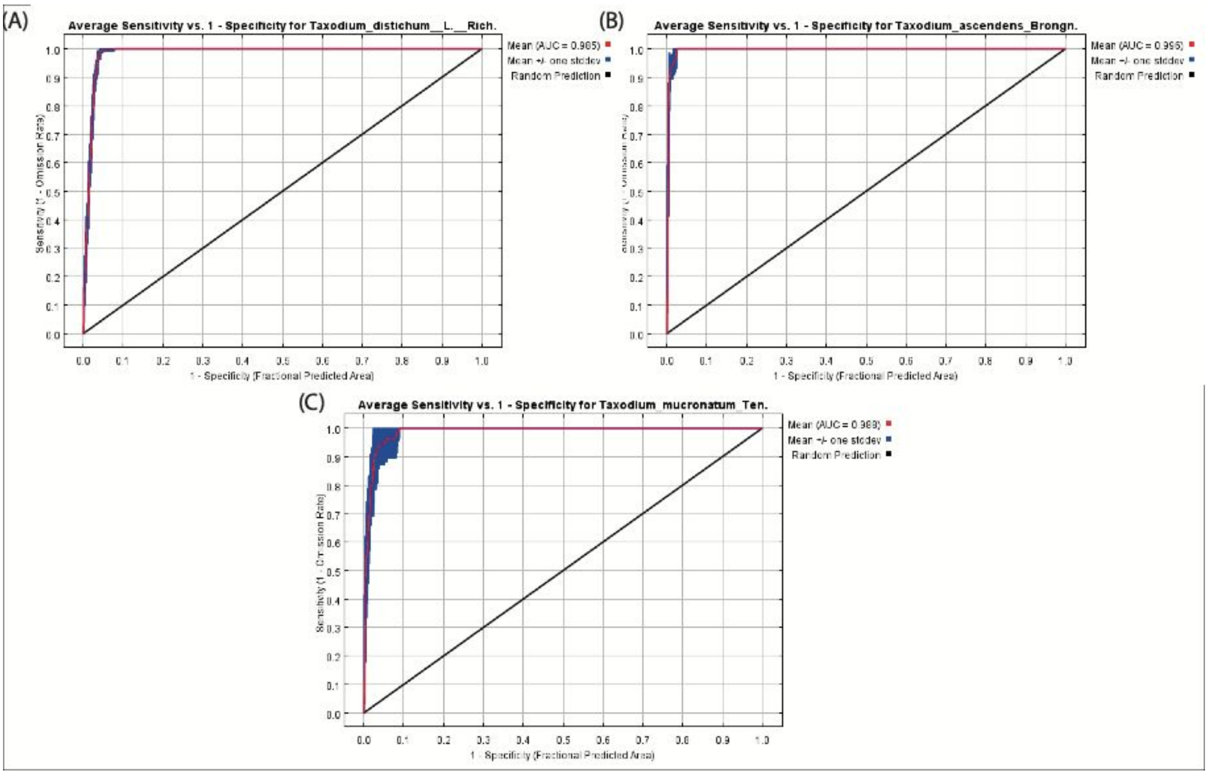

Figure S7. ROC plots for the three species models, with AUC values labelled top right. (A) *T. distichum*; (B) *T. ascendens*; (C) *T. mucronatum*,
