# Supplementary figures and images for: The revelation of genomic breed composition using target capture sequencing: a case of Taxodium
Source: For Res (Fayettev). 2024 Oct 8;4:e034. doi: 10.48130/forres-0024-0031 (PMC11524225; doi:10.48130/forres-0024-0031)

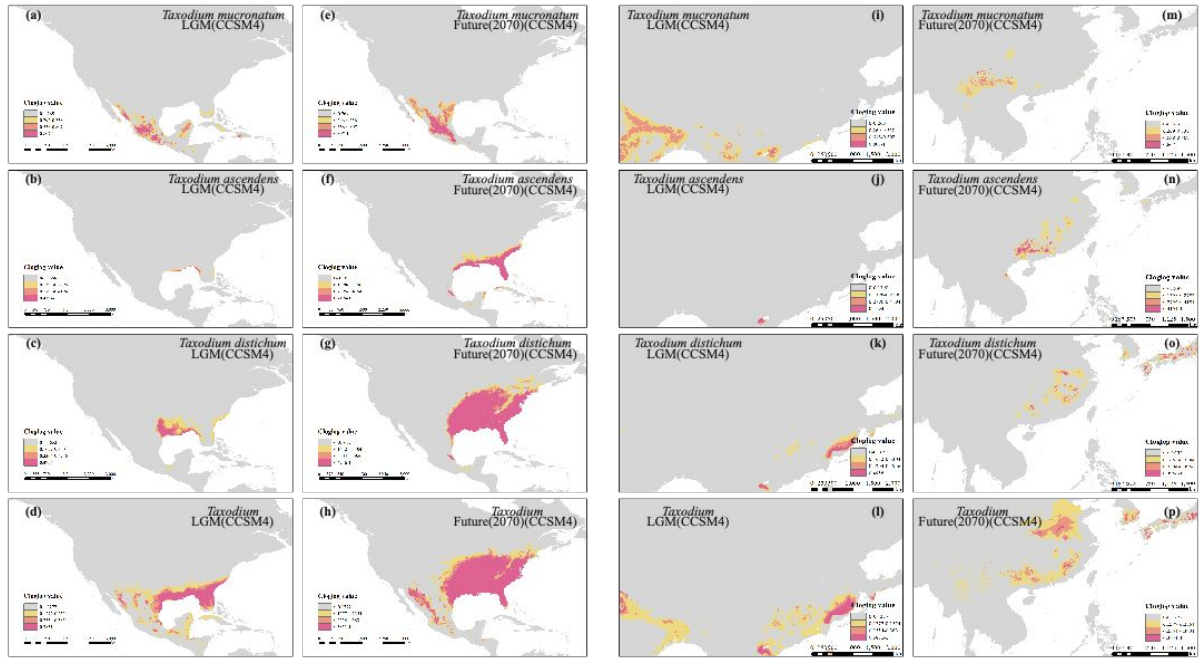

Figure S6. Species Distribution Modeling of the Three *Taxodium* Species in LGM and future 2070

Supplement: Supplementary file 1 — Supplementary data to this article can be found online. [file forres-0024-0031-S1.zip › 10.48130_forres-0024-0031-Suppl-FigureS6.pdf]
